# Risk Factors and Clinical Significance for Retrolisthesis of Adjacent Segment After Transforaminal Lumbar Interbody Fusion

**DOI:** 10.1111/os.70301

**Published:** 2026-04-02

**Authors:** Zhongmao Xu, Qinghua Zhao, Bin Wang, Zezhang Zhu, Yong Qiu, Xu Sun

**Affiliations:** ^1^ Division of Spine Surgery, Department of Orthopedic Surgery, Nanjing Drum Tower Hospital, Affiliated Hospital of Medical School Nanjing University Nanjing China

**Keywords:** adjacent segment disease, facet joint, paraspinal muscles, retrolisthesis, transforaminal lumbar interbody fusion

## Abstract

**Objective:**

Adjacent segment retrolisthesis is a common yet frequently overlooked complication after lumbar fusion, which may lead to reoperation. However, its risk factors, particularly those related to preoperative degenerative status and intraoperative variables, remain poorly understood. Therefore, this study aimed to elucidate the risk factors that contribute to the development of retrolisthesis in the adjacent segment following transforaminal lumbar interbody fusion (TLIF).

**Methods:**

We retrospectively reviewed 473 patients who underwent lower lumbar fusion for degenerative diseases from June 2017 to September 2022, with a minimum follow‐up of 2 years. Seventy patients who developed radiographic retrolisthesis postoperatively were included in the RR group, and 18 patients with symptoms were classified into the symptomatic retrolisthesis (SR) group. Using a 1:2 ratio, 140 patients without retrolisthesis were matched as the non‐retrolisthesis (NR) group. Preoperative MRI was used to assess fat infiltration and cross‐sectional area of the erector spinae, multifidus, and psoas muscles, as well as total endplate score and disc degeneration. CT was used to evaluate facet degeneration and pedicle screw‐related facet joint violation. Independent sample *t*‐tests and chi‐square tests were used for group comparisons, and multivariate logistic regression analysis was performed to identify independent risk factors.

**Results:**

Baseline age, sex, and bone mineral density were comparable between groups. Multivariate analysis showed that higher preoperative total endplate score (OR 2.086, 95% CI 1.496–2.907, *p* < 0.001), greater paraspinal muscle fat infiltration (OR 1.117, 95% CI 1.046–1.192, *p* = 0.001), facet degeneration (OR 2.838, 95% CI 1.762–4.570, *p* < 0.001), and postoperative facet violation (OR 1.911, 95% CI 1.330–2.746, *p* = 0.001) were independent risk factors for RR. Predictors of SR included total endplate score (OR 3.506, *p* = 0.002), fat infiltration of paraspinal muscles (OR 1.230, *p* = 0.008), facet degeneration (OR 8.940, *p* = 0.002), and postoperative facet violation (OR 2.873, *p* = 0.024).

**Conclusion:**

Preoperative degeneration of adjacent endplates, facet joints, and paraspinal muscles, together with postoperative facet joint violation, appears to be significantly associated with the development of retrolisthesis. Patients with retrolisthesis often present with persistent or severe low back pain after lumbar fusion, resulting in impaired quality of life.

## Graphical abstract

Preoperative degeneration of endplates, facet joints, and paraspinal muscles, along with intraoperative facet violation, are key predictors of retrolisthesis after TLIF, leading to worse pain and functional outcomes.

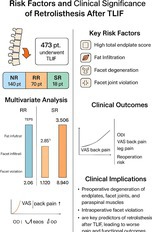

## 1 Introduction

Adjacent segment disease (ASD) is a well‐known complication after lumbar fusion surgery [1]. It may present as spinal canal stenosis, disc height reduction, anterolisthesis, or retrolisthesis [2]. Among these, retrolisthesis has been reported to occur in 20.9%–44% of cases [3, 4]. Its presence has been associated with persistent postoperative low back pain and an increased risk of subsequent reoperation after lumbar fusion [5, 6, 7]. Therefore, identifying the risk factors for postoperative retrolisthesis is of significant clinical importance for guiding postoperative rehabilitation and surgical decision‐making.

Previous studies have explored the association between retrolisthesis and sagittal alignment parameters, suggesting that retrolisthesis may occur as a compensatory mechanism to maintain global spinal balance [3, 5, 8]. However, there is a paucity of research on the roles of the disc, facet joints, paraspinal muscles, and ligamentous complex in the development of postoperative lumbar retrolisthesis. As the structural integrity and function of the lumbar spine primarily rely on the intervertebral discs, facet joints, and ligamentous support, disruption or degeneration of these components may compromise spinal stability [9, 10, 11]. Notably, pre‐existing degeneration in adjacent intervertebral discs, facet joints, paraspinal muscle atrophy or fatty infiltration, and intraoperative facet joint violation (FJV) have all been shown to adversely affect postoperative outcomes and increase the risk of complications. Therefore, it is essential to consider the condition of the musculoskeletal system, discs, and facet joints when evaluating the pathogenesis of retrolisthesis. Based on these considerations, this study was conducted with the following objectives: (i) To evaluate the association of preoperative degenerative changes in adjacent discs, facet joints, and paraspinal muscles, together with intraoperative variables, with postoperative retrolisthesis following transforaminal lumbar interbody fusion (TLIF); (ii) to determine the clinical significance of postoperative retrolisthesis.

## 2 Methods

### 2.1 Patients

After obtaining the approval from the ethics committee of our hospital (2025‐0452‐01), we retrospectively reviewed the clinical and radiological data of patients who had been treated with lower lumbar TLIF by a single surgical team (Dr. X.S.) from June 2017 to September 2022. Patients who met the following inclusion criteria were enrolled: (1) aged between 40 and 80 years at the time of surgery; (2) diagnosed with lumbar degenerative diseases; (3) fusion in the low lumbar region (L4‐S1); (4) a minimum follow‐up of 2 years; (5) complete set of radiological data, including preoperative MRI and lateral standing lumbar X‐rays, postoperative lateral standing lumbar X‐rays, and the latest follow‐up lateral standing lumbar X‐rays, MRI. The exclusion criteria were as follows: (1) history of spinal infection, tumor, trauma, or surgery; (2) preoperative concomitant lumbar scoliosis > 10°.

**FIGURE 1:**
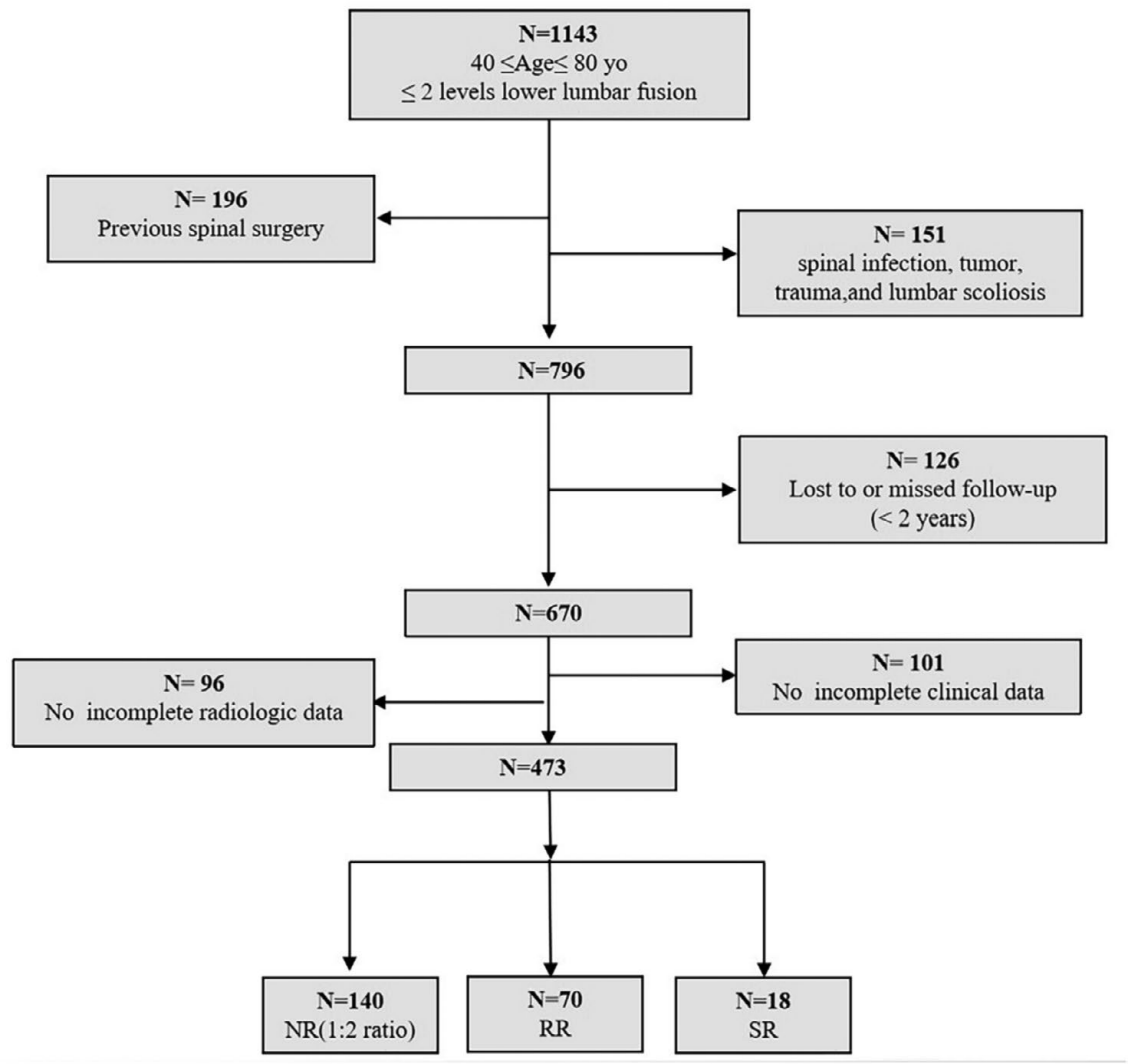
The algorithm of patient enrollment.

According to the criteria proposed by Wang et al. [2], radiological retrolisthesis (RR) is defined as a slip of one vertebral body by at least 3 mm in relation to the next caudal vertebral body on the upright neutral lateral radiograph at final follow‐up. Symptomatic retrolisthesis (SR) was defined as new clinical symptoms, such as low back pain, leg pain, numbness, or intermittent claudication that relapsed or deteriorated during the follow‐up period or necessitated reoperation. These symptoms were confirmed to be primarily related to radiologically evidenced retrolisthesis. A total of 473 patients who underwent TLIF surgery were retrospectively reviewed. Among them, 70 patients with new‐onset radiographic evidence of retrolisthesis were classified into the RR group. Eighteen patients who developed retrolisthesis accompanied by new‐onset lower limb symptoms or who required reoperation were further classified into the SR group. To identify potential risk factors for retrolisthesis, 140 patients without radiographic evidence of retrolisthesis were selected as the non‐retrolisthesis (NR) group using a 1:2 matching ratio with the RR group. Demographic and clinical data of all groups were retrospectively reviewed (Figure 1).

### 2.2 Radiological and Clinical Assessment

The following sagittal parameters were measured on standing full‐spine anteroposterior and lateral radiographs: thoracic kyphosis (TK); lumbar lordosis (LL); pelvic incidence (PI); pelvic tilt (PT); sacral slope (SS). CT scans were conducted with a spiral CT scanner (LightSpeed 16, GE Medical Systems, Milwaukee, WI, USA), and MRI scans were obtained using a 1.5‐T MRI system (Philips, Eindhoven, the Netherlands). Preoperative disc degeneration was assessed using the Pfirrmann classification system [12], which assigns grades from 1 to 5 based on T2WI. Preoperative facet degeneration (FD) was evaluated on CT and graded from 0 to 3 [13]. Postoperative FJV was subsequently assessed on CT and graded from 1 to 3, with the final score calculated by summing the superior FJV scores for both sides [14].

The degree of fat infiltration (DFF) and the cross‐sectional area of the erector, multifidus, and psoas muscles at the adjacent level were analyzed preoperatively using ImageJ software (version 1.48, National Institutes of Health, Bethesda, Maryland, USA). The total cross‐sectional area and average DFF of the paraspinal muscles were measured bilaterally.

Endplate scores were assigned for each endplate on T1WI, ranging from 1 to 6 points. The severity of endplate damage was classified according to the system established by Rajasekaran et al. [15]. The preoperative total endplate scores (TEPS) for the adjacent segment was calculated by summing the endplate scores of the lower endplate of the upper vertebra and the upper endplate of the lower vertebra, yielding a range of 2–12 [16, 17].

To verify the reliability of the FJV, 40 patients were randomly selected for assessment by three researchers (M.X., X.S., and Q.H). Each researcher was blinded to patient information to assess both inter‐ and intra‐observer reliability. The FJV demonstrated good intra‐ and inter‐observer reliability and reproducibility, with an intraclass correlation coefficient (ICC) of 0.833 and 0.845, respectively.

The preoperative, postoperative, and latest follow‐up Oswestry Disability Index (ODI) and Visual Analog Scale (VAS) scores for back and leg pain were used to assess clinical outcomes. Questionnaires were completed independently by patients before the operation and during the follow‐up.

### 2.3 Statistical Analysis

Statistical analyses were performed using SPSS version 27.0 (SPSS Inc., Chicago, IL, USA). Continuous variables were expressed as mean ± standard deviation. Independent sample *t*‐tests were used to compare disc degeneration, TEPS, DFF and total cross‐sectional area of paraspinal muscles, FJV, and FD between groups. The chi‐square test or Fisher's exact test was applied to analyze categorical variables, including comorbidities, gender, diagnosis, and lifestyle habits. Variables with a *p* value < 0.1 in the univariate analysis and those that passed the collinearity diagnostics were included in a logistic regression analysis to identify independent risk factors associated with retrolisthesis. The predictive performance of these risk factors was evaluated using the area under the receiver operating characteristic (ROC) curve. *p* value < 0.05 was considered statistically significant.

## 3 Results

### 3.1 Demographics

A total of 88 patients were diagnosed with retrolisthesis, including 70 in the RR group and 18 in the SR group. As shown in Table 1, the mean age at surgery was 54.9 ± 11.2 years in the RR group (24 males, 46 females) and 59.2 ± 8.0 years in the SR group (10 males, 8 females), with follow‐up durations of 28.9 ± 9.7 and 29.6 ± 1.0 months, respectively. The NR group included 140 patients (51 males, 89 females), with a mean age at surgery of 54.3 ± 11.0 years and a follow‐up duration of 27.1 ± 8.3 months. Although SR patients were slightly older than RR patients, the difference was not statistically significant (*p* = 0.075). No significant differences were found in BMI, BMD, smoking or alcohol history, number of fused segments, diagnosis, or comorbidities between groups.

**TABLE 1:** Characteristics of patients.

Characteristics	NR group (*n* = 140)	RR group (*n* = 70)	SR group (*n* = 18)	*p* ^1^	*p* ^2^
Age (years)	54.3 ± 11.0	54.9 ± 11.2	59.2 ± 8.0	0.731	0.075
Follow‐up time	44.4 ± 13.2	45.8 ± 16.1	46.2 ± 15.8	0.700	0.415
*T*‐score	−0.8 ± 1.7	−0.9 ± 1.6	−1.0 ± 1.8	0.801	0.201
BMI (kg/m^2^)	24.6 ± 2.6	24.1 ± 2.2	25.8 ± 2.6	0.23	0.077
Gender (female\male)	89\51	46\24	8\10	0.543	0.129
Comorbidities					
Diabetes	32	21	1	0.312	0.124
Hypertension	27	19	2	0.217	0.530
Fusion levels					
L4‐L5	61	34	7	0.783	0.167
L5‐S1	45	21	6		
L4‐S1	34	15	5		
Diagnosis					
Lumbar herniated disc	31	11	5	0.119	0.839
Lumbar spinal stenosis	38	13	5		
Spondylolisthesis	71	46	8		

*Note: p* ^1^: NR versus RR; *p* ^2^: NR versus SR.

Abbreviations: BMD = bone mineral density; BMI = body mass index.

### 3.2 Comparisons Between Groups

Table 2 demonstrates that the RR group exhibited significantly greater preoperative disc degeneration, FD, and more severe postoperative FJV compared to the NR group (all *p* < 0.05). Additionally, the RR group had a significantly higher TEPS (7.4 ± 1.5) than the NR group (5.9 ± 1.2, *p* < 0.05). Although there were no statistically significant differences in the cross‐sectional area of the erector, multifidus, or psoas muscles, nor in the total cross‐sectional area between the two groups (all *p* > 0.05), the DFF in the erector and multifidus muscles, as well as the average DFF of paraspinal muscles, was significantly higher in the RR group (all *p* < 0.05).

**TABLE 2:** Evaluation of preoperative and postoperative radiographic parameters.

Characteristics	NR group (*n* = 140)	RR group (*n* = 70)	SR group (*n* = 18)	*p* ^1^	*p* ^2^
TEPS	5.9 ± 1.2	7.4 ± 1.5	7.6 ± 1.3	0.001	0.001
Disc degeneration	3.3 ± 0.5	3.6 ± 0.5	3.6 ± 0.4	0.001	0.008
FD	0.8 ± 0.8	1.6 ± 0.8	2.0 ± 0.6	0.001	0.001
CSA of Psoas (cm^2^)	9.8 ± 4.6	10.3 ± 5.9	10.4 ± 4.8	0.619	0.630
CSA of erector (cm^2^)	19.0 ± 15.4	18.9 ± 14.0	19.5 ± 17.6	0.975	0.893
CSA of multifidus (cm^2^)	6.3 ± 4.9	7.8 ± 9.6	5.3 ± 4.8	0.238	0.446
Total CSA (cm^2^)	35.1 ± 23.4	37.0 ± 27.5	32.4 ± 22.9	0.662	0.658
DFF of Psoas (%)	5.8 ± 2.7	6.3 ± 3.9	6.6 ± 2.8	0.426	0.282
DFF of erector (%)	17.3 ± 8.7	22.5 ± 7.7	28.1 ± 7.5	< 0.001	< 0.001
DFF of multifidus (%)	20.6 ± 10.3	28.4 ± 9.4	33.2 ± 5.7	< 0.001	< 0.001
Average DFF (%)	14.1 ± 6.5	19.0 ± 5.7	21.8 ± 6.3	< 0.001	< 0.001
Postoperative FJV	0.7 ± 1.0	1.7 ± 1.3	2.1 ± 1.1	0.001	0.001
TK	24.2 ± 7.5	24.6 ± 8.2	22.6 ± 9.5	0.737	0.420
LL	46.0 ± 11.7	45.5 ± 13.4	46.2 ± 14.2	0.805	0.935
PI	43.6 ± 16.2	47.6 ± 14.6	48.5 ± 15.8	0.088	0.229
PT	16.5 ± 7.9	20.1 ± 13.4	18.2 ± 7.0	0.181	0.124
SS	33.9 ± 3.0	34.3 ± 2.9	34.6 ± 3.5	0.314	0.238

*Note: p* ^1^: NR versus RR; *p* ^2^: NR versus SR.

Abbreviations: CSA = cross‐sectional area; DFF = degree of fatty infiltration; FD = facet degeneration; FJV = facet joint violation; TEPS = total endplate score.

**FIGURE 2:**
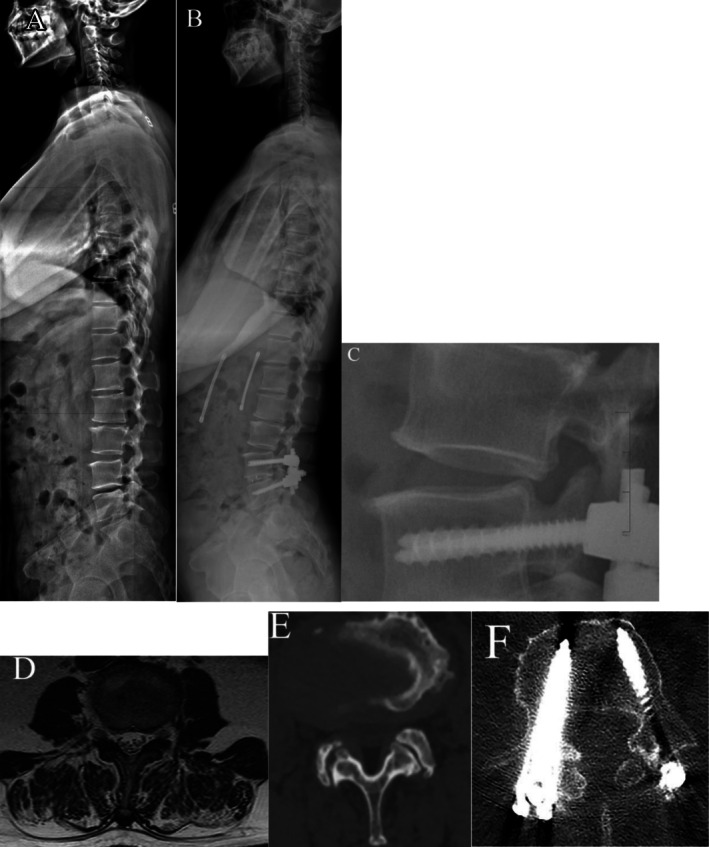
(A) A 47‐year‐old female patient diagnosed with L4/5 lumbar spinal stenosis who subsequently underwent L4/5 TLIF. (B, C) Standing lateral radiograph at 5‐year follow‐up demonstrates L3 retrolisthesis, accompanied by new‐onset lower limb symptoms. (D) Preoperative axial and sagittal T2‐weighted MRI at the L3/4 level reveal marked fatty infiltration of paraspinal muscle. (E, F) Preoperative CT demonstrates Grade 3 facet joint degeneration at L3/4, and postoperative CT confirms Grade 3 facet joint violation at the same level.

**FIGURE 3:**
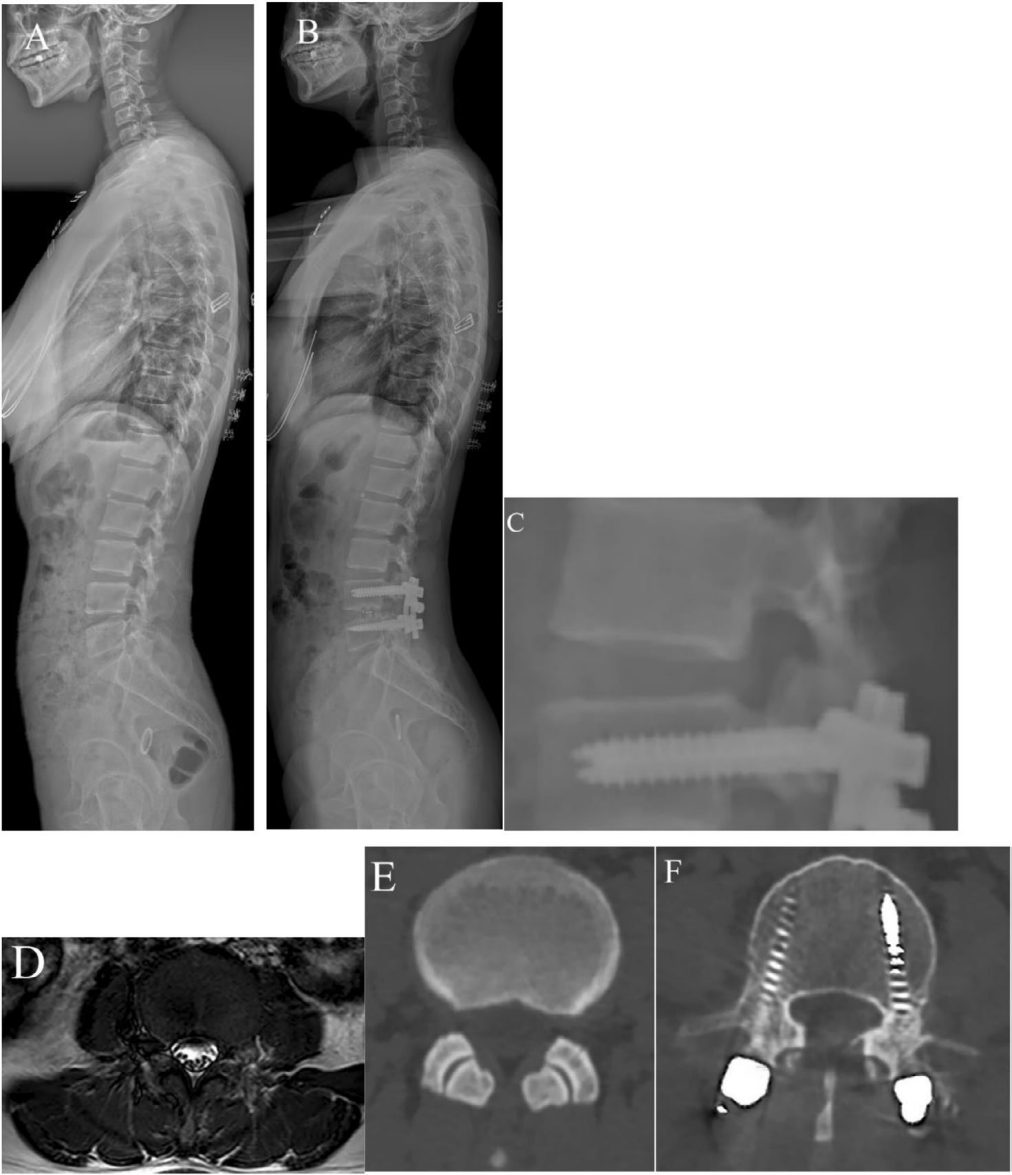
(A) A 48‐year‐old female patient diagnosed with L4/5 lumbar spinal stenosis. (B, C) Lateral radiograph at 3‐year follow‐up showing no radiographic evidence of degeneration at the adjacent L3/4 level. (D) Preoperative axial and sagittal MRI at L3/4 demonstrating well‐preserved paraspinal muscle morphology. (E, F) Preoperative CT shows no facet joint degeneration at L3/4, while postoperative CT reveals no facet joint violation at the same level.

Similarly, Table 2 shows that the SR group had significantly more severe preoperative disc degeneration, FD, and postoperative FJV than the NR group (all *p* < 0.05). The SR group also demonstrated significantly higher TEPS (7.6 ± 1.3) compared to the NR group (5.9 ± 1.2, *p* < 0.05). The DFF in the erector and multifidus muscles, as well as the average DFF of paraspinal muscles, was significantly greater in the SR group (all *p* < 0.05). However, the cross‐sectional area measurements of the erector, multifidus, and psoas muscles, as well as the total cross‐sectional area, did not differ significantly between the SR and NR groups (all *p* > 0.05). Representative cases are shown in Figures 2 and 3.

### 3.3 Multivariable Analysis for Retrolisthesis

**FIGURE 4:**
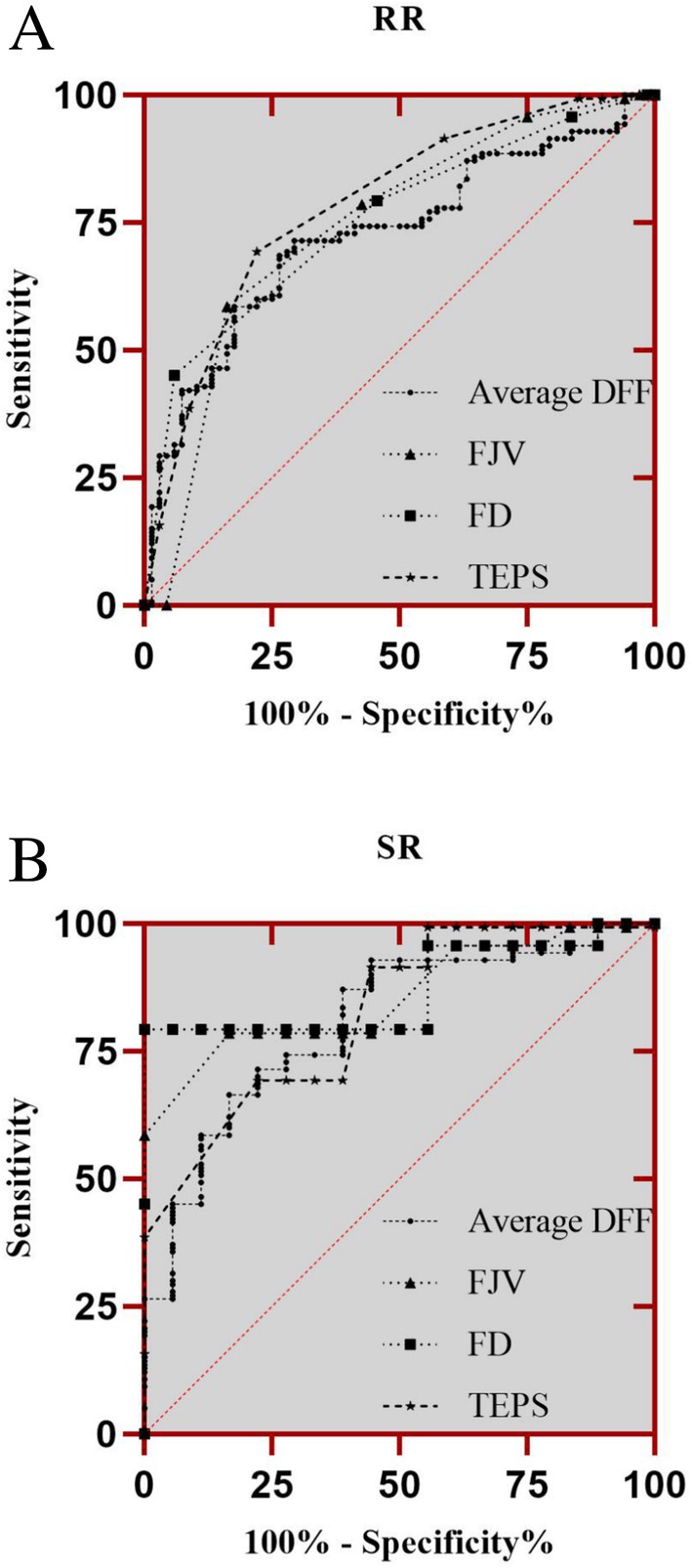
(A) Receiver operating characteristic (ROC) curve using the total endplate scores (TEPS), facet degeneration (FD), average degree of fatty infiltration (DFF) of paraspinal muscles, and postoperative facet joint violation (FJV) as a predictor of radiological retrolisthesis (RR). (B) ROC curve using the TEPS, FD, average DFF of paraspinal muscles and postoperative FJV as a predictor of symptomatic retrolisthesis (SR).

Multivariate logistic regression (Table 3) identified TEPS, fat infiltration of paraspinal muscle, FD, and postoperative FJV as independent predictors of RR (all *p* < 0.01). ROC analysis (Figure 4A) showed good discriminative ability, with AUCs of 0.784 (TEPS), 0.753 (FD), 0.743 (postoperative FJV), and 0.725 (fat infiltration of paraspinal muscle), respectively. Similarly, for SR, TEPS, fat infiltration of paraspinal muscle, FD, and postoperative FJV were also significant predictors (Table 4), with corresponding AUCs of 0.823, 0.871, 0.857, and 0.807, respectively (Figure 4B).

**TABLE 3:** Multivariate logistic regression analysis for potential risk factors of RR.

Variables	OR	95% CI	*p*
Average DFF	1.117	1.046–1.192	0.001
FD	2.838	1.762–4.570	< 0.001
Postoperative FJV	1.911	1.330–2.746	0.001
Disc degeneration	1.678	0.738–3.816	0.217
TEPS	2.086	1.496–2.907	< 0.001

Abbreviations: CI = confidence interval; DFF = degree of fatty infiltration; FD = facet degeneration; FJV = facet joint violation; OR = odds ratio; TEPS = total endplate score.

**TABLE 4:** Multivariate logistic regression analysis for potential risk factors of SR.

Variables	OR	95% CI	*p*
Average DFF	1.230	1.056–1.432	0.008
FD	8.940	2.181–36.649	0.002
Postoperative FJV	2.873	1.146–7.201	0.024
Disc degeneration	2.228	0.337–14.750	0.406
TEPS	3.506	1.592–7.721	0.002

Abbreviations: CI = confidence interval; DFF = degree of fatty infiltration; FD = facet degeneration; FJV = facet joint violation; OR = odds ratio; TEPS = total endplate score.

### 3.4 Quality of Life

Table 5 demonstrates that all three groups showed significant postoperative improvement in both VAS back pain and VAS leg pain compared to their respective preoperative scores (*p* < 0.05). There were no significant intergroup differences in either ODI, VAS back or leg pain scores at the preoperative or immediate postoperative assessments (*p* > 0.05). This indicates that the initial severity of pain was comparable between groups, and early postoperative pain relief was similarly achieved. Notably, at the latest follow‐up, the RR group reported significantly higher VAS back pain scores compared to the NR group (*p* < 0.05), and the SR group exhibited significantly worse ODI score and higher VAS score for both back and leg pain than the NR group. These findings suggest that postoperative retrolisthesis may have a detrimental effect on long‐term pain and functional outcomes after surgery.

**TABLE 5:** Clinical outcome evaluations.

Characteristics	NR group (*n* = 140)	RR group (*n* = 70)	SR group (*n* = 18)	*p* ^1^	*p* ^2^
VAS back pain					
Preoperation	6.4 ± 0.9	6.4 ± 1.1	6.6 ± 1.0	0.931	0.40
Postoperation	2.6 ± 0.8	2.7 ± 0.7	2.6 ± 0.5	0.424	0.905
Last‐follow up	1.4 ± 0.6	1.7 ± 0.6	6.1 ± 0.3	0.002	0.001
VAS leg pain					
Preoperation	5.8 ± 1.1	5.6 ± 0.8	5.8 ± 0.4	0.149	0.744
Postoperation	2.2 ± 0.8	2.0 ± 0.8	1.9 ± 0.5	0.156	0.079
Last‐follow up	0.8 ± 0.7	0.8 ± 0.7	5.4 ± 0.5	0.625	0.001
ODI scores					
Preoperation	44.3 ± 15.1	43.4 ± 13.6	48.1 ± 16.1	0.684	0.342
Postoperation	22.9 ± 9.9	23.8 ± 9.8	24.1 ± 9.3	0.559	0.658
Last‐follow up	20.6 ± 13.9	21.4 ± 12.6	43.3 ± 13.6	0.721	0.001

*Note: p* ^1^: NR versus RR; *p* ^2^: NR versus SR.

Abbreviations: ODI = Oswestry Disability Index; VAS = Visual Analog Scale.

## 4 Discussion

The present study investigated the risk factors associated with retrolisthesis in patients who had undergone lumbar fusion procedures. Our results demonstrate that preoperative fat infiltration of paraspinal muscles, TEPS, FJV, and FD are all independent risk factors for RR and SR. Patients with retrolisthesis may experience poorer surgical outcomes following fusion surgery.

### 4.1 Risk Factors for Retrolisthesis: Preoperative Degenerative Changes and Intraoperative Facet Violation

Previous studies have consistently reported a higher incidence of retrolisthesis in older patients, indicating a strong association between spinal degeneration and the onset of this condition. Additionally, the degeneration of adjacent facet joints, intervertebral discs, and paraspinal muscles has been established as a pivotal cause of ASD [9, 11]. As the primary manifestation of ASD, the occurrence of retrolisthesis is closely linked to these degenerative factors. In our study, FJV and FD were identified as significant risk factors for RR and SR in the lumbar spine. Facet joints play a crucial role in force distribution along the spine [18]. Preexisting FD and FJV may increase the likelihood of adjacent segment problems. It has been shown that FD and FJV contribute to the development of ASD [19]. Furthermore, FJVs can exacerbate FD and serve as a risk factor for ASD associated with FD [20, 21]. Therefore, it is essential to consider FD before surgery and take precautions to protect the facet joints during surgery.

Previous research has demonstrated the critical role of paraspinal muscles in spinal health and their significant association with the development of ASD. In our study, we found that a higher fat infiltration of the paraspinal muscles could serve as a predictor for RR and SR following spinal arthrodesis. Paraspinal muscle atrophy plays a key role in vertebral body changes and thoracolumbar pathology [16]. Fatty infiltration of paraspinal muscles can occur without changes in the total muscle size, making it an important indicator of muscle quality and functional status [22]. Han et al. [23] reported that fatty infiltration of the paraspinal muscles is a predictive factor for postoperative revision surgery and lower back pain. Similarly, Kim et al. [19] suggested that preexisting fatty degeneration of the paraspinal muscles is strongly associated with an increased risk of developing ASD.

Additionally, we found that an increase in the TEPS is associated with the occurrence of RR and SR. The vertebral endplate plays a key role in providing nutrition to the disc and adjacent tissues while alleviating pressure on the disc and lumbar spine [24]. Endplate degeneration may contribute to the risk of ASD. Harada et al. [25] suggested that endplate abnormalities could predict clinical outcomes following surgery. Furthermore, Li et al. [26] found that endplate defects may influence the development of slippage and lumbar destabilization. This suggests that endplate degeneration may accelerate the onset of retrolisthesis in adjacent segments.

### 4.2 Clinical Impact of Retrolisthesis on Postoperative Outcomes

RR is a common finding following lumbar fusion surgery and may progress to symptomatic ASD. As documented in previous studies, radiological ASD typically remains asymptomatic [2]. Despite being categorized as a subtype of radiological ASD, RR can progress to SR, potentially leading to new or more severe neurological symptoms. SR of adjacent fused segments can result in spinal stenosis and significant neural compression [5], which may profoundly affect surgical outcomes. In this study, patients in the RR group exhibited higher VAS scores for back pain at the final follow‐up compared to the control group. Additionally, ODI, VAS back pain, and VAS leg pain scores in the SR group were much higher than those in the control group at the last follow‐up. New neurological symptoms were observed in the SR group, while the RR group reported persistent residual back pain. Both conditions were associated with poorer postoperative recovery and may indicate a less favorable surgical prognosis. Consequently, spine surgeons must maintain vigilance in clinical practice to avert potential complications.

### 4.3 Clinical Implications

This study underscores the importance of comprehensive preoperative assessment and meticulous surgical planning in preventing postoperative retrolisthesis following lumbar fusion. Identifying and managing modifiable risk factors can reduce the incidence of segmental instability and enhance long‐term clinical outcomes. During surgery, surgeons should carefully preserve the integrity of posterior spinal elements, minimize unnecessary FJV, and adopt individualized strategies—such as minimally invasive techniques—for patients with poor paraspinal muscle quality or advanced degenerative changes. Furthermore, early recognition and intervention for radiographic retrolisthesis may help prevent its progression to symptomatic forms and subsequent neurological compromise. Overall, these findings emphasize the value of a holistic, patient‐specific approach to optimize surgical efficacy and mitigate the risk of adjacent segment deterioration.

### 4.4 Strengths and Limitations

This study has several strengths. First, the relatively large sample size provides sufficient power to identify independent risk factors. Second, the comprehensive assessment of multiple factors offers a holistic understanding of retrolisthesis pathogenesis. Third, the inclusion of both radiographic and SR allows characterization of the full clinical spectrum, from asymptomatic imaging findings to clinically significant disease.

However, several limitations should be acknowledged. Firstly, the SR group was relatively small, which may limit the statistical power and stability of the multivariate analysis; accordingly, the associated odds ratios with wide confidence intervals should be interpreted with caution. Second, the relatively short mean follow‐up duration captures only early‐to‐mid‐term postoperative retrolisthesis, whereas ASD is a long‐term dynamic process; therefore, some patients in the NR group may develop retrolisthesis with longer follow‐up. In addition, this study is subject to the inherent limitations of a retrospective design, which may introduce selection bias, and unmeasured confounding cannot be fully excluded due to reliance on available recorded data. Therefore, the observed associations may have been influenced by unmeasured variables. Future studies with larger sample sizes, longer follow‐up durations, and prospective designs are warranted to further validate these findings. Finally, although sagittal alignment parameters were measured, none showed significant intergroup differences on univariate analysis; this is acknowledged as a potential limitation given the established role of global sagittal imbalance in retrolisthesis.

In conclusion, high average fat infiltration, endplate degeneration, FJV, and FD are independently associated with retrolisthesis. Moreover, retrolisthesis is associated with persistent or severe lower back pain, adversely affecting postoperative recovery and quality of life.

## Author Contributions

Conception and design: Xu Sun and Yong Qiu. Administrative support: Qinghua Zhao, Xu Sun, Bin Wang, Zezhang Zhu, and Yong Qiu. Provision of study materials or patients: Qinghua Zhao, Xu Sun, Bin Wang, Zezhang Zhu, and Yong Qiu. Collection and assembly of data: Zhongmao Xu, Qinghua Zhao, and Xu Sun. Data analysis and interpretation: Zhongmao Xu, Qinghua Zhao, and Xu Sun. Manuscript writing: All authors. Final approval of manuscript: All authors.

## Funding

This work was supported by Jiangsu Provincial Medical Innovation Center of Orthopedic Surgery (CXZX202214) and Development Project of Nanjing Science and Technology Commission and Foundation (ZKX20020).

## Disclosure

The authors have nothing to report.

## Ethics Statement

The study was conducted in accordance with the Declaration of Helsinki and was approved by the Ethics Committee of Nanjing Drum Tower Hospital, Affiliated Hospital of Medical School, Nanjing University (no. 2025‐0452‐01) on June 05, 2025, with the need for written informed consent waived.

## Conflicts of Interest

The authors declare no conflicts of interest.

## Data Availability

The data that support the findings of this study are available on request from the corresponding author. The data are not publicly available due to privacy or ethical restrictions.
